# Full fusion of proximal thoracic curve helps to prevent postoperative cervical tilt in Lenke type 2 adolescent idiopathic scoliosis patients with right-elevated shoulder

**DOI:** 10.1186/s12891-017-1730-y

**Published:** 2017-08-23

**Authors:** Jun Jiang, Bang-ping Qian, Yong Qiu, Bin Wang, Yang Yu, Ze-zhang Zhu

**Affiliations:** The Department of Spine Surgery, the Affiliated Drum Tower Hospital of Nanjing University Medical School, Zhongshan Road 321, Nanjing, 210008 China

**Keywords:** Cervical tilt, Adolescent idiopathic scoliosis, Shoulder elevation

## Abstract

**Background:**

To date, no study had reported the phenomenon of deteriorated postoperative cervical tilt in Lenke type 2 adolescent idiopathic scoliosis patients. The purpose of this study is to evaluate the cervical tilt in Lenke type 2 adolescent idiopathic scoliosis patients with right-elevated shoulder treated by either full fusion or partial/non fusion of the proximal thoracic curve.

**Methods:**

A total of 30 Lenke type 2 AIS patients with preoperative right-elevated shoulder underwent posterior spinal instrumentation from 2009 to 2011 were included in this study. All the subjects were divided into 2 groups according to the selection of upper instrumented vertebra. There were 14 cases proximally fused to T1 or T2 (Group A) and 16 cases proximally fused to T3 or below (Group B). Both standing anteroposterior and sagittal X-ray films of the spine obtained preoperatively, one week after the operation, and at a minimum of two-year follow-up were analyzed with respect to the following parameters: cervical tilt, T1 tilt, proximal thoracic Cobb angle, main thoracic Cobb angle, apical vertebral translation of proximal thoracic curve, apical vertebral translation of main thoracic curve, radiographic shoulder height, cervical lordosis, proximal thoracic kyphosis and main thoracic kyphosis.

**Results:**

Most (83.3%) of the patients in these two groups gained satisfactory shoulder balance after surgery. However, the cervical tilt significantly improved in group A (*p* < 0.001) but deteriorated in group B (*p* < 0.001). In group A, the decrease of cervical tilt significantly positively correlated with that of T1 tilt (*p* < 0.001). In group B, the increase of cervical tilt significantly positively correlated with both the increase of T1 tilt (*p* < 0.001) and the increase of apical vertebral translation of proximal thoracic curve (*p* < 0.05).

**Conclusions:**

Lenke type 2 AIS patients with right-elevated shoulder gain improved shoulder but deteriorated cervical tilt after partial/non fusion of proximal thoracic curve. Full fusion of proximal thoracic curve helps to prevent the residual cervical tilt in these patients.

## Background

Nowadays, it had been widely accepted that whether the proximal thoracic (PT) curve should be fused in adolescent idiopathic scoliosis (AIS) patients depended on both the flexibility of PT curve and the directionality of preoperative shoulder height [1, 2]. The PT curve had poor flexibility in Lenke type 2 AIS patients with low spontaneous correction ability in case of sole correction of main thoracic (MT) curve [3]. Therefore, both PT curve and MT curve should be fused if the Lenke type 2 AIS patient had a preoperative left-elevated shoulder since sole correction of the right MT curve could further elevate the left shoulder, which might lead to deterioration of the shoulder imbalance [4, 5]. On the contrary, if the patient had a preoperative right-elevated shoulder, fusion of PT curve was unnecessary because the right higher shoulder could be compensated by the left shoulder elevation gained from MT correction [6].

In our practice, we found that most of the Lenke type 2 patients with right-elevated shoulder had satisfactory shoulder balance after partial or non fusion of the PT curve. However, some patients complained of postoperative deteriorated cervical tilt, which had not been taken into account before surgery. To our best knowledge, no study has investigated the reasons for the deteriorated cervical tilt in Lenke type 2 AIS patients without PT curve fully fused. This study aims to evaluate the postoperative cervical tilt in AIS patients with double thoracic curve treated by either full fusion or partial/non fusion of the PT curve and to analyze the mechanism underlying this phenomenon with the purpose of aiding spine surgeons with preoperative planning.

## Methods

### Subjects

A total of 556 AIS patients underwent posterior pedicle screw instrumentation from July 2009 to November 2011 in our institution were retrospectively reviewed. The inclusion criteria were: 1) with Lenke type 2 curve (double thoracic curve); 2) with preoperative right-elevated shoulder; 3) with a minimum follow-up of 2 years. Finally, a total of 30 cases met the criteria mentioned above were included. All the patients were further divided into 2 groups according the selection of upper instrumented vertebra (UIV). Full fusion of PT curve was defined as proximal fusion to T2 or above, partial fusion of PT curve was defined as proximal fusion to T3 and non fusion of PT curve was defined as proximal fusion to T4 or below. There were 14 patients (12 females and 2 males) proximally fused to T1 (9 cases) or T2 (5 cases) in full fusion group (Group A) with an average age of 16.1 years (range, 12–20 years). The average PT curve was 44.7° with the flexibility of 19.2% and the average MT curve was 51.7° with the flexibility of 44.7% in Group A. There were 16 patients (14 females and 2 females) proximally fused to T3 (10 cases) or T4 (6 cases) in partial or non fusion group (Group B) with an average age of 15.1 years (range, 11–19 years). The mean PT curve was 42.3° with the flexibility of 18.4% and the mean MT curve was 60.3° with the flexibility of 41.2% in Group B. All these subjects were followed up for a mean of 2.8 years (2 years to 4 years). This study was approved by the institutional review board in our hospital.

### Radiographic evaluation

**Fig. 1:**
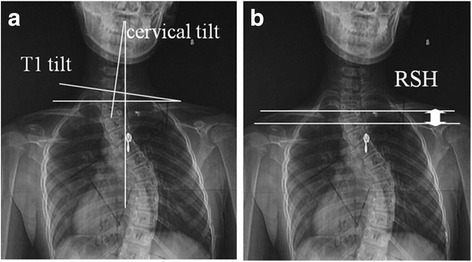
**a** and **b** Cervical tilt: the angle is formed by the intersection of the *vertical line* and the longitudinal axis of the cervical spine (**a**); T1 tilt: angle between the *line* along the superior endplate of T1 and a perpendicular to the *horizontal line* (**a**); Radiographic shoulder height (RSH): the difference in the soft tissue shadow directly superior to the acromioclavicular joint (the value is defined as positive when left shoulder is up and negative when right shoulder is up) (**b**)

**Fig. 2:**
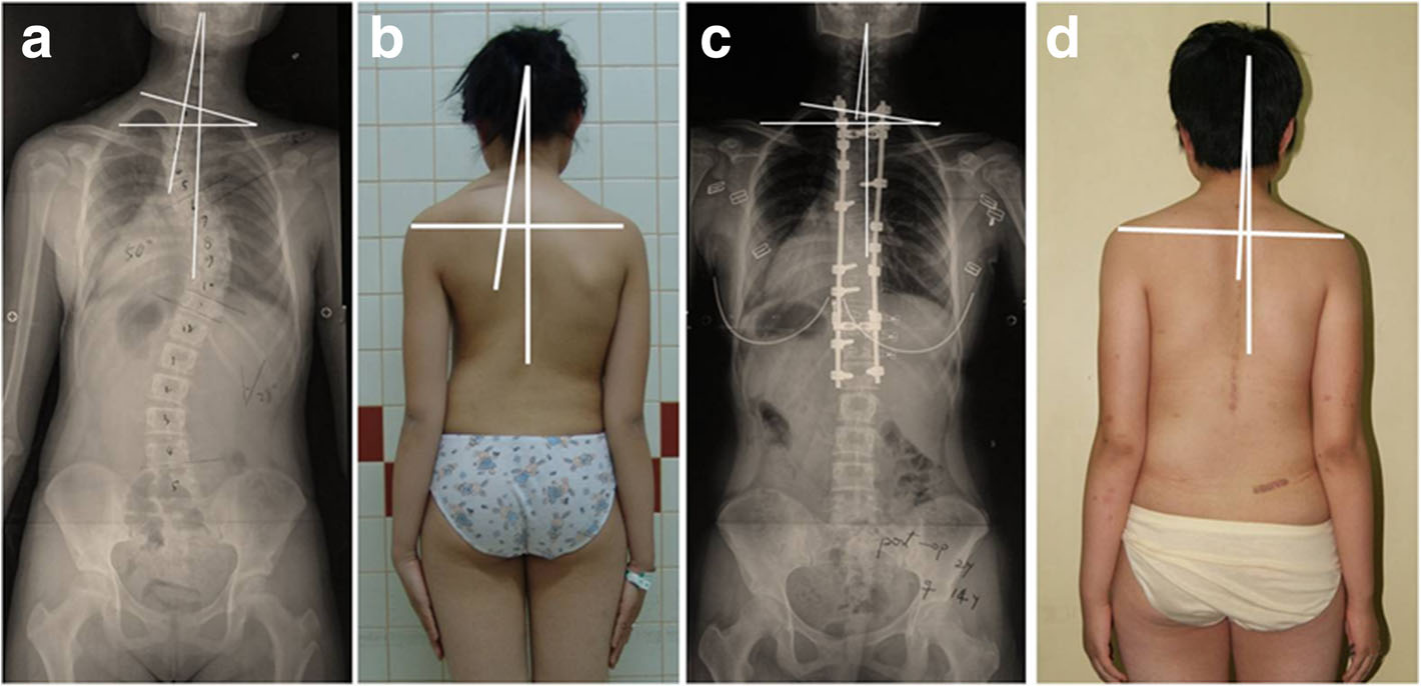
**a**-**d**. A 12 years old female patient with preoperative right-elevated shoulder fused to T1 level at the time of surgery (**a** and **b**). The cervical tilt was corrected from 13° (**a**) preoperatively, to 6° at the last follow-up (**c**). The T1 tilt decreased from 16° preoperatively, to 9° at the last follow-up (*p* < 0.001). The RSH changed from −1 mm (**a**) preoperatively, to 7 mm at the last follow-up (**c**). This patient had both good cervical balance and shoulder balance after the fusions of both PT and MT curves (**c** and﻿ **d**)

**Fig. 3:**
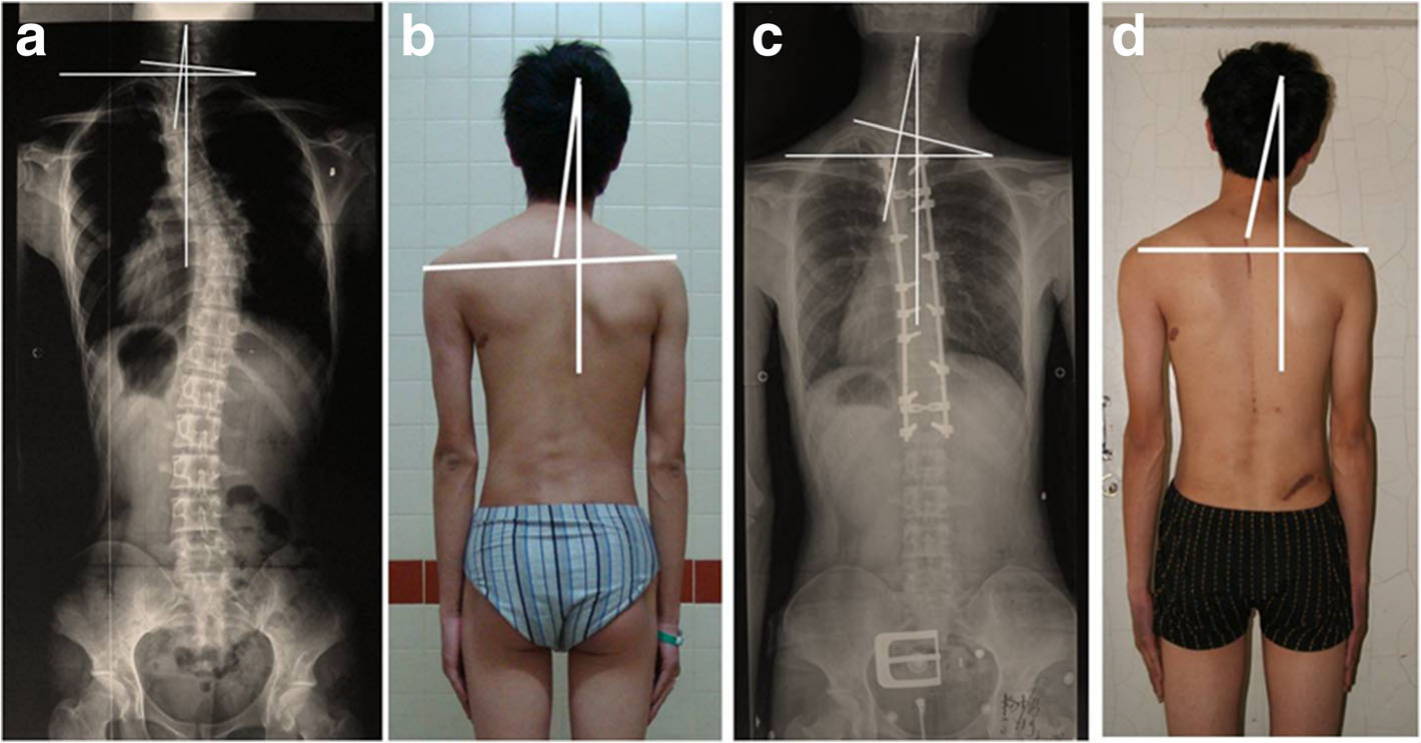
**a**-**d**. A 19 years old male patient with preoperative right-elevated shoulder fused to T3 level at the time of surgery (**a** and **b**). The cervical tilt increased from 4° (**a**) preoperatively to 11° at the last follow-up (**c**). The T1 tilt increased from 6° preoperatively, to 13° at the last follow-up (*p* < 0.001). The RSH changed from −7 mm (**a**) preoperatively, to 0 mm at the last follow-up (**c**). This patient had improved shoulder balance but deteriorated cervical balance after the partial fusion of the PT curve (**c** and **d**)

Both long-cassette standing anteroposterior and sagittal radiographs of the spine were obtained in all the subjects preoperatively, one week after surgery and at the last follow-up for measurements. All the subjects were requested to be in a natural standing posture with both the neck and shoulder fully relaxed when taking the anteroposterior radiographic examination and in a natural standing posture with bilateral arms straightly forward when taking the sagittal radiographic examination. The parameters assessed included the following: (1) cervical tilt [7]: the angle between the line following the longitudinal axis of the cervical spine and the vertical line (Fig. 1a); (2) T1 tilt: angle between a line along the superior endplate of T1 and a perpendicular to the horizontal line (Fig. 1a); (3) PT Cobb angle [8]; (4) MT Cobb angle [8]; (5) PT apical vertebral translation (AVT) [8]: the horizontal distance from the coronal centre of the apical vertebrae (the intersection of lines connecting the superior lateral corners of the vertebral body to the contralateral inferior lateral corners) to the C7 plumbline; (6) MT AVT [8]; (7) radiographic shoulder height (RSH) [9]: the difference in the soft tissue shadow directly superior to the acromioclavicular joint (Fig. 1b), (8) cervical lordosis (CL): the sagittal Cobb angle between the lower endplate of C2 and that of C7, (9) proximal thoracic kyphosis (PTK): the sagittal Cobb angle between the upper endplate of T2 and lower endplate of T5 [8]; and (10) main thoracic kyphosis (MTK): the sagittal Cobb angle between the upper endplate of T5 and lower endplate of T12 [8]. All the 3 sagittal parameters (CL, PTK and MTK) were defined as positive if the angles were kyphotic and negative if the angles were lordotic. The RSH was defined as positive when the left shoulder was higher and negative when the right shoulder was higher. Postoperative shoulder imbalance was defined as RSH more than 10 mm [9].

### Statistical Anaylsis

Statistical analysis was performed by SPSS 13.0 software (Chicago, IL, USA). The radiographic parameters measured one week after the operation and at the last follow-up were compared with those measured preoperatively by the paired-t test in each group. Correlation analysis was used to determine a Pearson coefficient (r) between the change of cervical tilt and the changes of the other parameters at the last follow-up in each group respectively. A value of *P* < 0.05 was considered to be statistically significant.

## Results

### Full fusion group (group a, Table 1)

The average PT curve was corrected from 44.7° preoperatively, to 15.8° after the operation (*p* < 0.001) and to 18.5° at the last follow-up (*p* < 0.001). The average MT curve was corrected from 51.7° preoperatively, to 15.6°after the operation (*p* < 0.001) and to 17.2° at the last follow-up (*p* < 0.001). The mean PT AVT decreased from 9.2 mm preoperatively, to 4.1 mm after the operation (*p* < 0.001) and 4.8 mm at the last follow-up (*p* < 0.05). The mean MT AVT decreased from 28.1 mm preoperatively, to 4.3 mm after the operation (*p* < 0.001) and 5.0 mm at the last follow-up (*p* < 0.001). The average RSH was changed from −4.6 mm preoperatively, to 4.9 mm after the operation (*p* < 0.001) and to 5.5 mm at the last follow-up (*p* < 0.001). The rate of postoperative shoulder imbalance was only 14.3% (2/14) at last follow-up. None of these 3 sagittal parameters was significantly changed both after the operation and at last follow-up (*p* > 0.05).

**Table 1:** Surgical outcomes in patients with PT curve fused (Group A, *n* = 14)

	Preoperative	Postoperative	Last follow-up
Cervical tilt(°)	9.6 ± 2.6	4.0 ± 1.4^a^	5.0 ± 3.5^a^
T1 tilt(°)	12.3 ± 3.4	5.8 ± 2.6^a^	6.6 ± 4.7^a^
PT curve(°)	44.7 ± 8.5	15.8 ± 6.7^a^	18.5 ± 7.9^a^
MT curve(°)	51.7 ± 11.8	15.6 ± 7.5^a^	17.2 ± 6.2^a^
PT AVT(mm)	9.2 ± 4.0	4.1 ± 4.0^a^	4.8 ± 4.5^a^
MT AVT(mm)	28.1 ± 7.8	4.3 ± 5.0^a^	5.0 ± 5.3^a^
RSH(mm)	−4.6 ± 3.5	4.9 ± 7.3^a^	5.5 ± 5.7^a^
CL	0.6 ± 11.1	−1.7 ± 9.3	−1.1 ± 9.0
PTK	13.9 ± 4.3	15.4 ± 3.7	14.8 ± 2.9
MTK	16.7 ± 7.7	18.1 ± 6.3	18.3 ± 4.4

*PT*: proximal thoracic curve, *MT*: main thoracic curve, *AVT*: apical vertebrae translation, *RSH*: radiographic shoulder height, *CL*: cervical lordosis, *PTK*: proximal thoracic kyphosis, *MTK*: main thoracic kyphosis * means the difference is statistically significant

The mean cervical tilt was improved from 9.6° preoperatively, to 4.0° after the operation (*p* < 0.001) and to 5.0° at the last follow-up (*p* < 0.001). The average T1 tilt decreased from 12.3 ° preoperatively, to 5.8° after the operation (*p* < 0.001) and to 6.6° at the last follow-up (*p* < 0.001).

### Partial/non fusion group (group B, Table 2)

The average PT curve was changed from 42.3° preoperatively, to 29.5° after the operation (*p* < 0.001) and to 31.6° at the last follow-up (*p* < 0.001). The average MT curve was corrected from 60.3° preoperatively, to 22.5° (*p* < 0.001) after the operation and to 24.0° at the last follow-up (*p* < 0.001). The mean PT AVT increased from 4.2 mm preoperatively, to 9.5 mm (*p* < 0.001) after the operation and 9.7 mm at the last follow-up (*p* < 0.001). The mean MT AVT decreased from 43.3 mm preoperatively, to 12.5 mm (*p* < 0.001) after the operation and 16.0 mm at the last follow-up (*p* < 0.001). The average RSH was changed from −12.4 mm preoperatively, to 2.0 mm after the operation (*p* < 0.001) and to 2.1 mm at the last follow-up (*p* < 0.001). The incidence of postoperative shoulder imbalance was only 18.7% (3/16) at the last follow-up. None of these 3 sagittal parameters was significantly changed both after the operation and at last follow-up (*p* > 0.05).

**Table 2:** Surgical outcomes in patients with PT curve partial/non fused (Group B, *n* = 16)

	Preoperative	Postoperative	Last follow-up
Cervical tilt(°)	5.4 ± 2.1	10.9 ± 3.1^a^	10.5 ± 2.5^a^
T1 tilt(°)	7.2 ± 2.5	14.7 ± 2.5^a^	13.7 ± 2.7^a^
PT curve(°)	42.3 ± 8.5	29.5 ± 6.7^a^	31.6 ± 6.3^a^
MT curve(°)	60.3 ± 12.4	22.5 ± 7.1^a^	24.0 ± 7.5^a^
PT AVT(mm)	4.2 ± 3.2	9.5 ± 3.7^a^	9.7 ± 3.6^a^
MT AVT(mm)	43.3 ± 13.3	12.5 ± 9.3^a^	16.0 ± 10.0^a^
RSH(mm)	−12.4 ± 8.3	2.0 ± 4.1^a^	2.1 ± 4.7^a^
CK	−0.6 ± 7.5	−2.7 ± 9.3	−2.4 ± 9.1
PTK	12.7 ± 3.8	13.6 ± 4.1	13.4 ± 3.2
MTK	16.4 ± 9.7	18.5 ± 8.8	17.3 ± 7.9

*PT*: proximal thoracic curve, *MT*: main thoracic curve, *AVT*: apical vertebrae translation, *RSH*: radiographic shoulder height, *CL*: cervical lordosis, *PTK*: proximal thoracic kyphosis, *MTK*: main thoracic kyphosis. ^a^ means the difference is statistically significant

The mean cervical tilt was deteriorated from 5.4° preoperatively, to 10.9° after the operation (*p* < 0.001) and to 10.5° at the last follow-up (*p* < 0.001). The average T1 tilt increased from 7.2 ° preoperatively, to 14.7° after the operation (*p* < 0.001) and to 13.7° at the last follow-up (*p* < 0.001).

### Correlation analysis (Table 3)

In Group A, the decrease of cervical tilt was significantly positively correlated with that of T1 tilt at the last follow-up (*p* < 0.001). In Group B, the increase of cervical tilt was significantly positively correlated with both that of T1 tilt and that of PT AVT at the last follow-up (*p* < 0.05).

**Table 3:** Correlations of the change of cervical tilt with those of other parameters

	Group A (r)	Group B (r)
T1 change	0.830^a^	0.762^a^
PT curve change	0.331	−0.164
MT curve change	0.386	−0.184
PT AVT change	0.113	0.545^a^
MT AVT change	−0.141	0.134
RSH change	−0.497	0.127
CK change	−0.252	0.097
PTK change	0.371	−0.159
MTK change	0.045	−0.299

*PT*: proximal thoracic curve, *MT*: main thoracic curve, *AVT*: apical vertebrae translation, *RSH*: radiographic shoulder height, *CL*: cervical lordosis, *PTK*: proximal thoracic kyphosis, *MTK*: main thoracic kyphosis. ^a^ means statistically significant

## Discussions

One of the main goals of the surgical treatment of AIS patients is to gain the satisfactory cosmetic appearance, such as shoulder balance [10, 11]. Proper recognition and treatment of PT curve in AIS patients is a primary factor in preventing residual shoulder unbalance. Several previous studies have recommended fusions of both PT and MT curves in the patients with a significant structural PT curve (Lenke type 2 curve) since the untreated rigid PT curve has low ability to compensate the correction gained in the MT curve and increases the risk of shoulder derangement after the sole correction of MT curve [2]. However, nowadays it has been increasingly recognized that a structural PT curve does not always necessarily imply the fusions of both PT and MT curves [6]. The preoperative shoulder height is another important factor in determining the whether the PT curve should be included in the fusion range. Actually, the preoperative shoulder height is diverse (left-elevated shoulder, leveled shoulder and right-elevated shoulder) in Lenke type 2 patients. Patients with right-elevated shoulder had either a larger MT Cobb angle or a more proximal apex of MT curve when compared with those with leveled or left-elevated shoulder [12]. Partial or non fusion of PT curve has been advocated in these patients with preoperative right-elevated shoulder since the correction of MT curve will elevate the left shoulder, which helps to restore the shoulder balance after surgery [6, 13].

Although the previous literatures are rich regarding the influence of upper instrumented vertebra (UIV) on postoperative shoulder balance in AIS patients, no study specifically concerning on such influence on postoperative cervical tilt has been documented. Following the current treatment guidelines [6, 13], we started treating Lenke type 2 patients with preoperative right higher shoulder by the strategy of selective thoracic fusion (partial or non fusion of PT curve) from the latter half of 2010. In this study, 16 Lenke type 2 patient (Group B) with preoperative right-elevated shoulder had the PT curve partial/non fused and most of them had satisfactory shoulder balance after the sole correction of the MT curve. However, these patients had substantial deteriorated cervical tilt, which had not been predicted when making the surgical plan. Unlike the patients in Group B, most of the patients in Group A gained both good shoulder and cervical balance after the fusion of PT curve, indicating that the fusion of PT curve might help to maintain the cervical balance in these AIS patients (Fig. 2). Therefore, the cervical balance seems to be independent of the shoulder balance. Kwan also found that the phenomenon of cervical tilt was distinct from that of shoulder imbalance with poor correlation with each other [14, 15]. However, to date, the mechanism underlying deteriorated cervical tilt in Lenke type 2 AIS patients without PT curve fully fused has not been investigated.

It has been known that the spontaneous correction of unfused PT curve is closely related to the amounts of correction obtained in MT curve in AIS patient [16]. Previous studies had reported the overcorrection of MT curve beyond the flexibility of PT curve, resulting in the deterioration of spinal imbalance. With the introduction of more powerful instrumentation, such as segmental pedicle screw that further enhances the MT correction, the risk of PT curve decompensation substantially increases. A rigid structural PT curve (Lenke type 2 curve) has low ability to accommodate the correction gained from the MT curve, which further increases such risk when it is left unfused. The PT curve flexibility was only 19.2% in group A and 18.4% in group B, respectively. Actually, the untreated PT curve of the patients in Group B was decompensated after the sole correction of MT curve, which was reflected by both the increase of T1 tilt and that of PT AVT. The correlation analysis demonstrated that both the increase of T1 tilt and that of PT AVT were significantly positively associated with the increase of cervical tilt. Since the increases of T1 tilt and PT AVT were the reflections of PT curve decompensation, this result suggested that it was the decompensation of the rigid PT curve resulted from sole correction of MT curve that led to the deterioration of cervical tilt (Fig. 3). Different from the patients in Group B, the patients in Group A gained both shoulder and cervical balance after the correction of PT curve. The decrease of the cervical tilt was found to be significantly associated with the decrease of T1 tilt, indicating that the more corrections T1 tilt, the more correction of cervical tilt. All the data confirm that the full fusion of PT curve with the correction of T1 tilt helps to prevent the deterioration of cervical tilt in Lenke type 2 patients. Although all the cases in this study had preoperative right higher shoulder, they were not statistically homogeneous between Group A and Group B. Patients in group A had larger cervical tilt, T1 tilt, RSH but smaller MT curve than those in group B. Therefore, no radiographic parameter was compared between these 2 groups by independent-t test in our study. However, two different change tendencies of cervical tilt between 2 groups with different surgical strategy were demonstrated in our study by paired-t test in each group, respectively.

**Fig. 4:**
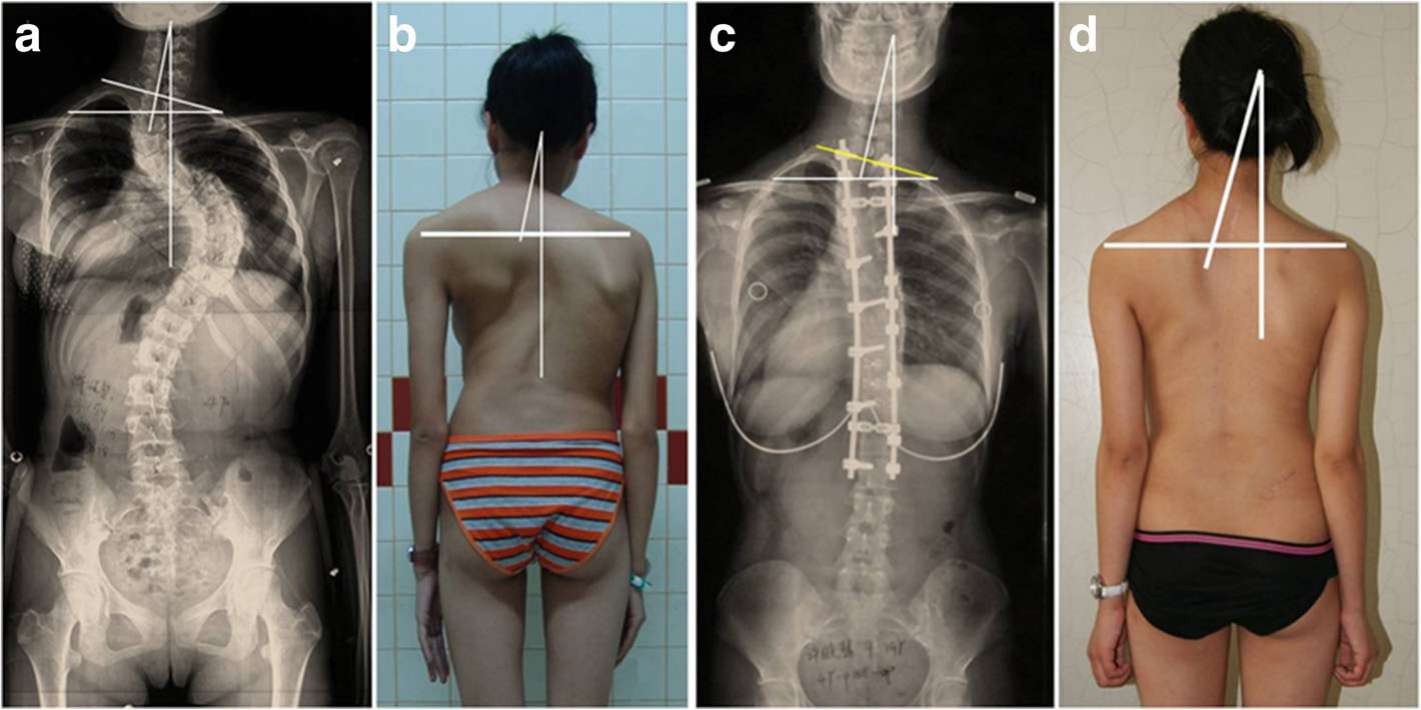
**a**-**d**. A 15 years old female patient with preoperative right-elevated shoulder fused to T1 level at the time of the operation (**a** and **b**). The cervical tilt increased from 14° (**a**) preoperatively, to 16° at the last follow-up (**c**). The T1 tilt increased from 15° preoperatively, to 22° at the last follow-up (*p* < 0.001). The RSH changed from −6 mm (**a**) preoperatively, to 6 mm at the last follow-up (**c**). Although the patient underwent the fusions of both PT curve and MT curve, she still had the residual cervical tilt since the T1 tilt was not well corrected when fusing the PT curve (**c** and **d**)

It was noticeable that the change of T1 tilt was significantly associated with that of cervical tilt in both two groups. T1 can be considered as the “ground base” of the cervical vertebrae. Maintaining a horizontal T1 might help to restore the coronal cervical balance in AIS patients. In one patient in Group A, although the PT curve had been fully fused, the cervical tilt still existed after the operation since the T1 tilt was not well corrected (Fig. 4). Therefore, when fusing the PT curve, we advise that compressive force should be applied on the convex side of the PT curve to achieve a horizontal T1.

Besides the coronal cervical tilt, the sagittal cervical alignments of Lenke type 2 AIS patients after surgery were also evaluated in our study. The sagittal cervical alignment is highly associated with the sagittal thoracolumbar alignment in AIS patients [17]. It had been found that the pedicle screw or hybrid instruments had a hypokyphotic effect on thoracic spine, which could lead to the residual cervical kyphosis [18, 19]. However, no sagittal parameter was found to be significantly changed after surgery in our patients. We presume that it might be attributed to the proper choice of correction strategy during the surgery, which helps to maintain the thoracic kyphosis in AIS patients. Furthermore, no association was founded between the change of cervical tilt and those of 3 sagittal parameters in both 2 groups, indicating that the coronal cervical balance may be independent of the sagittal cervical alignment in these patients.

Several limitations of this study should be mentioned in this study. Firstly, the follow-up of our patients averaged 2.8 years with the maximum of 4 years. Whether the cervical tilt could be spontaneous compensated or not at the time of longer follow-up remained unknown. Secondly, the sample size was relatively small in our study. The preliminary results in this study need further study with larger sample size to confirm. Thirdly, this is only a radiographic study without the patient’s self-assessment/satisfaction evaluated. Lastly, the radiographic parameters are different from cosmetic parameters in AIS patients. The change of cosmetic cervical tilt in Lenke type 2 patients after surgery needs the next study to investigate. However, the postoperative cervical tilt after sole correction of MT curve at the medium follow-up still deserves the spine surgeons’ special attentions when making the surgical plan.

## Conclusion

This current study demonstrates that Lenke type 2 AIS patients with right-elevated shoulder gain improved shoulder balance but deteriorated cervical tilt after partial/non fusion of PT curve. Full fusion of the PT curve with the correction of T1 tilt helps to avoid the deterioration of the cervical tilt in these patients. The changes of cosmetic parameters related to neck and shoulder balance after surgery and the effects of such changes on quality of life in these patients are needed to be investigated in the next study.

## Abbreviations

AIS: adolescent idiopathic scoliosis
AVT: apical vertebral translation
CL: cervical lordosis,
MT: main thoracic
MTK: main thoracic kyphosis.
PT: proximal thoracic;
PTK: proximal thoracic kyphosis
RSH: radiographic shoulder height
